# Activation of GABA_B_ Receptor Suppresses Diabetic Neuropathic Pain through Toll-Like Receptor 4 Signaling Pathway in the Spinal Dorsal Horn

**DOI:** 10.1155/2018/6016272

**Published:** 2018-12-10

**Authors:** Peng Liu, Hong-Bin Yuan, Shuang Zhao, Fei-Fei Liu, Yu-Qing Jiang, Yue-Xian Guo, Xiu-Li Wang

**Affiliations:** ^1^Department of Anesthesiology, The Third Hospital of Hebei Medical University, No. 139 Ziqiang Road, Shijiazhuang 050051, China; ^2^Department of Anesthesiology, Changzheng Hospital Affiliated to Second Military Medical University, No. 415 Fengyang Road, Shanghai 200003, China

## Abstract

Diabetic neuropathic pain (DNP) is a prevalent complication in diabetes patients. Neuronal inflammation and activation of Toll-like receptor 4 (TLR4) are involved in the occurrence of DNP. However, the underlying mechanisms remain unclear. Downregulation of gamma-aminobutyric acid B (GABA_B_) receptor contributes to the DNP. GABA_B_ receptor interacts with NF-*κ*B, a downstream signaling factor of TLR4, in a neuropathic pain induced by chemotherapy. In this study, we determined the role of TLR4/Myd88/NF-*κ*B signaling pathways coupled to GABA_B_ receptors in the generation of DNP. Intrathecal injection of baclofen (GABA_B_ receptor agonist), LPS-RS ultrapure (TLR4 antagonist), MIP (MyD88 antagonist), or SN50 (NF-*κ*B inhibitor) significantly increased paw withdrawal threshold (PWT) and paw withdrawal thermal latency (PWTL) in DNP rats, while intrathecal injection of saclofen (GABA_B_ receptor blocker) decreased PWT and PWTL in DNP rats. The expression of TLR4, Myd88, NF-*κ*Bp65, and their downstream components IL-1 and TNF-*α* was significantly higher in the spinal cord tissue in DNP rats compared to control rats. Following inhibition of TLR4, Myd88, and NF-*κ*B, the expression of IL-1 and TNF-*α* decreased. Activation of GABA_B_ receptors downregulated the expression of TLR4, Myd88, NF-*κ*Bp65, IL-1, and TNF-*α*. Blockade of GABA_B_ receptors significantly upregulated expression of TLR4, Myd88, NF-*κ*Bp65, IL-1, and TNF-*α*. These data suggest that activation of the TLR4/Myd88/NF-*κ*B signaling pathway is involved in the occurrence of DNP in rats. Activation of GABA_B_ receptor in the spinal cord may suppress the TLR4/Myd88/NF-*κ*B signaling pathway and alleviate the DNP.

## 1. Introduction

Diabetes mellitus is one of the most prevalent diseases that plagues more than 425 million people worldwide. Diabetic neuropathy, characterized by intractable pain and/or progressive sensory loss, is a common complication of diabetes mellitus caused by long-term diabetic damage [1]. Diabetic neuropathic pain (DNP) is one form of diabetic neuropathy caused by damaged nerves. The symptoms of DNP include paresthesia, hyperalgesia, allodynia, and spontaneous pain. Treating DNP is clinically challenging because the pain is difficult to describe [2, 3]. The underlying mechanisms of DNP are complex, but changes in central sensitization, including synaptic plasticity and imbalance of excitatory and inhibitory neurotransmitters in the spinal cord, play important roles in the process of the DNP [4–6].

Recent studies on the innate immune system and neuronal inflammation have provided insights into the role of neuronal inflammation in neuropathic pain [7]. Toll-like receptors 4 (TLR4), a member of TLR family and mainly mediate innate immune, are widely distributed in immune cells and microglial in the central nervous system. In addition to recognizing exogenous bacterial and fungal components, such as LPS, TLR4 bands to endogenous ligands such as high mobility group box-1 protein (HMGBl) and HSP60. After being activated, TLR4 activates 2 downstream signal pathways including Myd88-dependent or independent pathways. Myd88-dependent pathway is mainly mediated by TLR4/Myd88/NF-*κ*B activation and cytokine production [8]. The microglial Toll-like receptor 4 (TLR4) in the spinal cord is involved in initiating and maintaining microglial activation and neuronal inflammation [9–13]. The activation of TLR4 signaling pathway plays an important role in the occurrence and development of neuropathic pain caused by nerve injury or chemotherapy. Accordingly, the upregulation of TLR4 contributes to the development of the DNP, but the underlying mechanisms remain unclear [14].

We previously demonstrated that downregulation of GABA_B_ receptors participated in the occurrence of the DNP. GABA_B_ receptor activation rebalances excitatory and inhibitory neurotransmission and alleviated nociception [15, 16]. In paclitaxel-induced neuropathic pain, activation of GABA_B_ receptors influenced excitatory receptors and inhibited NF-*κ*B and downstream inflammatory factors, thus relieving neuronal inflammation [17]. Activation of GABA_B_ receptors downregulated TLR4-induced NF-*κ*Bp65 nuclear expression and release of proinflammatory cytokines in mixed glia of a multiple sclerosis (MS) model [18]. Collectively, these findings suggest that both GABA_B_ receptors and TLR4/NF-*κ*B signaling pathways play important roles in the occurrence of DNP.

In this study, we aimed to explore the role of TLR4/NF-*κ*B pathways in the formation of DNP. We further investigated the interactions between GABA_B_ receptor and the TLR4/NF-*κ*B signaling pathway in a DNP animal model to elucidate the relationship between GABA_B_ receptors and neuronal inflammation in DNP.

## 2. Materials and Methods

### 2.1. Ethics Statement

All experimental procedures were carried out in accordance with the National Institutes of Health Guide for the Care and Use of Laboratory Animals. The study was approved by the Animal Use Committee of Hebei Medical University. All behavioral tests and drug administrations were carried out during the dark phase.

### 2.2. Diabetic Neuropathic Pain Animal Model

Male SD rats (provided by animal experiment center, Hebei Medical University) aged 3 weeks old and weighing 150–170 g were used in this study. The rats were kept at 20–25°C with a normal light-dark cycle and free access to food and water after 3 days of adaptation. A total of 100 rats were randomly assigned to induce DNP as reported previously [4]. Briefly, the rats were fasted for 12 hours followed by peritoneal injection (i.p.) of STZ (60 mg/kg, Sigma) freshly dissolved in 0.1 mol/l citric acid buffer (pH 4.5) or were used as nondiabetic controls. After 7 days, blood samples were extracted from the tail vein of rats and blood glucose levels were measured using a blood glucose analyzer (Roche, Germany). Blood glucose level ≥ 16.7 mmol/l was considered hyperglycemic and deemed successful for establishment of diabetes. The paw withdrawal threshold (PWT) was measured 2 weeks after STZ injection according to the protocol of Dixon [19]. Blood glucose level > 16.7 mmol/l and PWT < 6 g were used as the cut-off criteria for successful model of DNP. DNP was successfully induced in 72 rats. Twelve rats were injected i.p. with equal volume of saline as normal controls.

### 2.3. Catheter Implantation

Nineteen days after STZ injection, DNP rats and normal rats were anesthetized with 3% pentobarbital sodium (1 ml/kg, i.p.) and a sterile polyethylene catheter (PE-10, Instech Laboratories Incorporation, Plymouth Meeting, PA, USA) was inserted into the gap between the L4/L5 vertebrae with the tip of the catheter located in the subarachnoid space. Two days after recovery from surgery, the rats were injected with 2% lidocaine (10 *μ*l) through the catheter to confirm that the catheter tip was in the subarachnoid space. Reversible hindlimb paralysis after injection was considered as a sign of successful catheter implantation.

### 2.4. Drug Treatment

**Figure 1:**
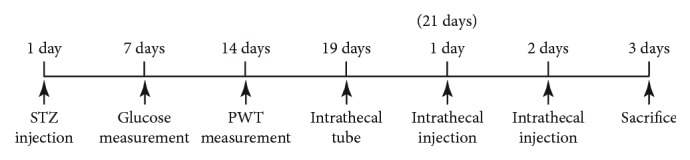


A total of 72 DNP rats and 12 normal control rats were successfully implanted with catheters and were injected with drugs intrathecally 2 days later. DNP rats were randomly divided into six groups with 12 rats per group: (1) the DNP group: 20 *μ*l saline (i.t.); (2) the DNP + baclofen group: baclofen (GABA_B_ receptor agonist, 0.5 *μ*g in 20 *μ*l, i.t.); (3) the DNP + saclofen group: saclofen (GABA_B_ receptor blocker, 30 *μ*g in 20 *μ*l, i.t.); (4) the DNP + LPS-RS group: LPS-RS ultrapure (a selective TLR4 antagonist, 20 *μ*g in 20 *μ*l, i.t.); (5) the DNP + MIP group: 500 *μ*M (Myd88 antagonist, 500 *μ*M in 20 *μ*l, i.t.); and (6) the DNP + SN50 group: SN50 (NF-*κ*B inhibitor, 200 ng in 20 *μ*l, i.t.). Rats received intrathecal injections once a day for 3 days. The 12 normal rats were treated with saline as the control group (control group). After each injection, artificial cerebrospinal fluid (ACSF, 10 *μ*l) was injected immediately to drain residual liquid in the catheter. ACSF comprises of the following components (in mmol/l): KCl 3.35, NaCl 138.6, MgCl_2_·6H_2_O 1.16, CaCl_2_·2H_2_O 1.26, NaH_2_PO4·2H_2_O 0.58, NaHCO_3_ 21.0, and glucose 10.0 (pH 7.4) Figure 1.

### 2.5. Assessment of Mechanical Allodynia and Thermal Hyperalgesia

Mechanical allodynia and thermal hyperalgesia were assessed by determining the mechanical PWT (Von Frey, Stoelting Co., Wood Company, USA) and paw withdrawal thermal latency (PWTL) (PL-200, Chengdu Taimeng Software Technology Company, China). PWT was measured 30 min after intrathecal injection. PWTL was measured 2 h after the PWT measurement.

### 2.6. PWT

The rats were placed in a transparent glass case which was in sound-proof place for 30 minutes. The PWT was measured using von Frey filaments applied vertically to the plantar surface of both hind paws to bend the filament for 6 s. Brisk withdrawal or paw flinching was deemed to be a positive response. As the response occurred, the next lower force was applied. If not, the next greater force was applied. The plantar stimulus producing a 50% withdrawal response was calculated by using the “up-down” method [19].

### 2.7. PWTL

The thermal radiation was applied to the plantar surface of both hind paws and the latency from start of radiation to paw withdrawal recorded. The thermal stimulus was repeated five times at an interval of five minutes, and the average value of final three tests was considered as PWTL [4].

### 2.8. Western Blot Assay

After 3 days of intrathecal injection, lumbar (L1–5) spinal dorsal horn of each rat was collected to assess the expression of TLR4, Myd88, and NF-*κ*B by Western blotting as reported previously [20]. Briefly, the spinal dorsal horn was lysed with RIPA buffer for 30 min at 4°C. Samples were centrifuged for 30 min at 12,000 rpm at 4°C. The supernatant proteins were collected. Proteins (40 *μ*g) were electrophoresed in 10% SDS polyacrylamide gels and transferred to polyvinylidene fluoride (PVDF) membranes. Membranes were blocked with 5% dried fat-free milk for 2 hours at room temperature and incubated overnight at 4°C with different primary rabbit polyclonal antibodies: *β*-actin (1 : 2000, rabbit anti-rat, ABclonal, USA), TLR4 (1 : 300, rabbit anti-rat, Abcam, USA), Myd88 (1 : 500, rabbit anti-rat, Arigobio, Taiwan, ROC), and NF-*κ*Bp65 (1 : 500, rabbit anti-rat, Arigobio, Taiwan, ROC). The membranes were treated with horseradish peroxidase-conjugated goat anti-rabbit immunoglobulin G antibody (1 : 4000, PTG, USA). Proteins were visualized using UVP gel imaging system (UVP, USA). The intensity of immunoreactive bands was analyzed by the Quantity One 4.6 software (Bio-Rad, USA). *β*-actin served as an internal control. Relative protein expression = (optical density value of the object protein)/(optical density value of *β*-actin).

### 2.9. Immunofluorescence

After three days of injection and behavioral tests, rats were euthanized to collect tissues for histological analyses. Rats were anesthetized and transcardially perfused with warm saline followed by 4% cold paraformaldehyde. The lumbar (L1–5) spinal cord was collected and fixed in 4% paraformaldehyde immediately for 12 hours, and then cryoprotected in 30% sucrose solution, embedded in optimum cutting temperature compound, and sliced at thickness of 15 *μ*m. Sections were incubated in 10% H_2_O_2_ for 10 minutes. After blocking with 10% goat serum for 2 hours at room temperature, sections were incubated overnight at 4°C with rabbit polyclonal antibodies against TLR4 (1 : 100 Abcam), Myd88 (1 : 100 Arigobio), and NF-*κ*Bp65 (1 : 100 Arigobio). Sections were incubated with goat anti-rabbit secondary antibodies conjugated to Cy2 (1 : 100, Abcam, Cambridge, UK) for 2 hours at room temperature the next day. Expressions of TLR4, Myd88, and NF-*κ*B in the spinal cord dorsal horn were observed under an inverted fluorescent microscope (Olympus, Japan). Nuclei were dyed by DAPI; the blue granules were considered to be cell nucleus. Cells with green granules were identified as positively stained. For one indicator, five slides of the dorsal horn from each rat were chosen for quantification of positively stained cells in the spinal cord dorsal horn. For each slides, 5 visual fields were separately counted. One visual field was in the center of the slides. The other four visual fields were just adjacent to but not overlap with it.

### 2.10. ELISA

Following the last injection and behavioral tests, spinal dorsal horns of some rats in the seven groups were collected and homogenized to measure the expression of TNF-*α* and IL-1 by ELISA as described previously [21]. The spinal cord was collected, homogenized, and centrifuged at 2000*g* at 4°C for 20 min. The levels of TNF-*α* and IL-1 in the spinal cord were quantified by ELISA kit (ABclonal, USA). The absorbance (A) was detected at 450 nm (A450) as per manufacturer's instructions. The content of TNF-*α* and IL-1 in each sample was calculated according to the standard curve.

### 2.11. Statistical Analysis

Data were analyzed by the SPSS (version 17.0) software and were expressed as mean ± SEM. The results of time course measurement for the PWT and the PWTL were analyzed by two-way analysis of variance (ANOVA) followed by the Newman-Keuls post hoc test. The result analysis of Western blot, immunofluorescent staining, and ELISA was performed by one-way ANOVA. A *p* value < 0.05 was considered statistically significant.

## 3. Results

### 3.1. Mechanical and Thermal Withdrawal Threshold

Compared with those of the control group, mechanical PWT and PWTL of rats in the DNP, DNP + SN50, DNP + MIP, DNP + LPS-RS, DNP + baclofen, and DNP + saclofen groups were significantly decreased at 14 days and 21 days. Compared with baseline values, PWT and PWTL values in the DNP, DNP + SN50, DNP + MIP, DNP + LPS-RS, DNP + baclofen, and DNP + saclofen groups were significantly decreased at 14 days and 21 days. Rats in these groups met the diagnosis of DNP after i.p. injection of STZ (*p* < 0.05, Tables 1 and 2).

### 3.2. Effects of TLR4/NF-*κ*B Signal Pathway Inhibition on Mechanical PWT and PWTL

**Figure 2:**
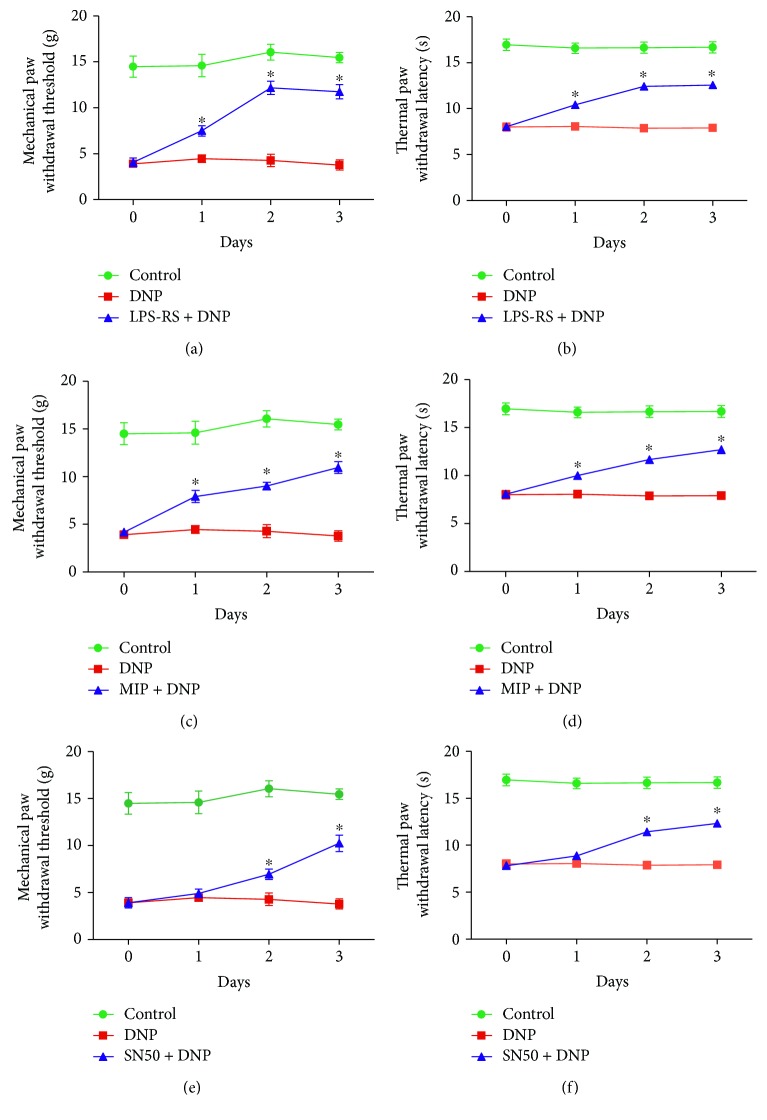
Neuropathic pain behavior in the control, diabetic neuropathic pain (DNP), DNP + LPS-RS (TLR4 antagonist), DNP + MIP (Myd88 antagonist), and DNP + SN50 (NF-*κ*B inhibitor) groups at 0, 1, 2, and 3 days after intrathecal administration. (a, c, e). The mechanical paw withdrawal threshold (PWT) (g). (b, d, f). The paw withdrawal thermal latency (PWTL) (s). Bar graphs represent mean ± SEM. ^∗^*p* < 0.05, compared with the DNP group (*n* = 12).

In the DNP + LPS-RS and DNP + MIP groups, inhibition of TLR4 with LPS-RS ultrapure or Myd88 with MIP significantly increased mechanical PWT and PWTL compared with those in the DNP group (saline) at 1, 2, and 3 days after i.t. injection (*p* < 0.05, *n* = 12). In the DNP + SN50 group, inhibition of NF-*κ*B (SN50) significantly attenuated mechanical and thermal allodynia compared with that in the DNP group at 1, 2, and 3 days after i.t. injection (*p* < 0.05, *n* = 12, Figure 2).

### 3.3. Effects of GABA_B_ Receptor Activation or Inhibition on Mechanical PWT and PWTL

**Figure 3:**
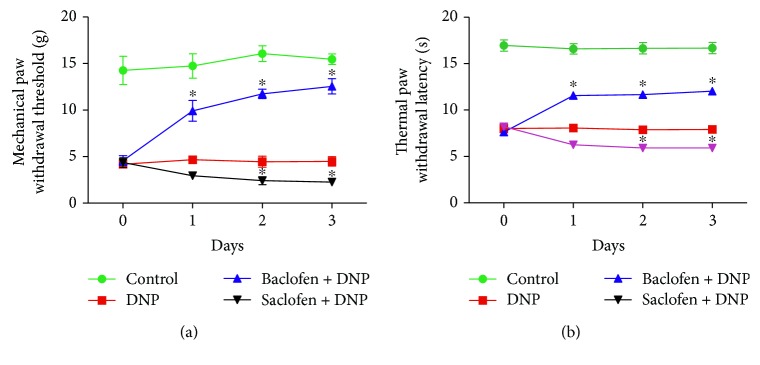
The development of mechanical allodynia and thermal hyperalgesia in the control, diabetic neuropathic pain (DNP), DNP + baclofen (GABA_B_ receptor agonist), and DNP + saclofen (GABA_B_ receptor blocker) groups at 0, 1, 2, and 3 days after intrathecal administration. (a) The mechanical paw withdrawal threshold (PWT). (b) The paw withdrawal thermal latency (PWTL). Bar graphs represent mean ± SEM. ^∗^*p* < 0.05, compared with the DNP group (*n* = 12).

In the DNP + baclofen group, activation of GABA_B_ receptors with baclofen significantly increased mechanical PWT and PWTL compared with those in the DNP group (saline) at 1, 2, and 3 days after i.t. injection (*p* < 0.05, *n* = 12). In the DNP + saclofen group, blockade of GABA_B_ receptors with saclofen further aggravated mechanical allodynia and thermal hyperalgesia compared with those in the DNP group (saline) at 1, 2, and 3 days after i.t. injection (*p* < 0.05, *n* = 12, Figure 3).

### 3.4. Effects of TLR4/NF-*κ*B Signal Pathway Inhibition on Myd88 and NF-*κ*B Expression

**Figure 4:**
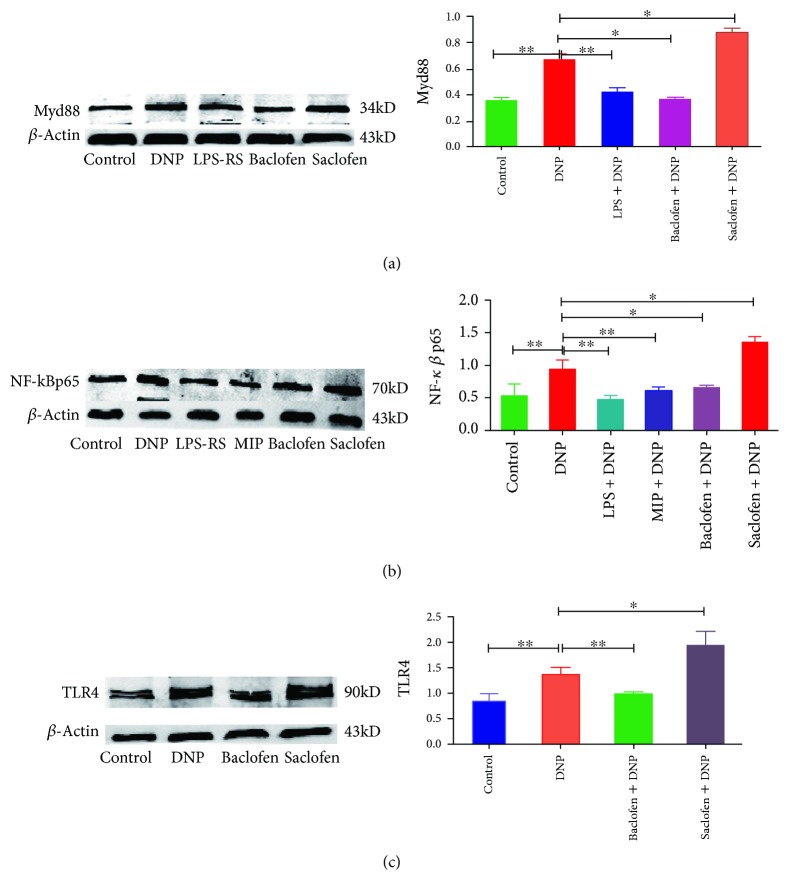
The protein expression of Myd88, TLR4, and NF-*κ*Bp65 in each group. (a) Myd88 in the control (*n* = 4), DNP (*n* = 4), DNP + LPS-RS (TLR4 antagonist, *n* = 6), DNP + baclofen (GABA_B_ receptor agonist, *n* = 4), and DNP + saclofen (GABA_B_ receptor blocker, *n* = 4) groups. (b) NF-*κ*Bp65 in the control (*n* = 4), DNP (*n* = 4), DNP + LPS-RS (TLR4 antagonist, *n* = 6), DNP + MIP (Myd88 antagonist, *n* = 6), DNP + baclofen (GABA_B_ receptor agonist, *n* = 4), and DNP + saclofen (GABA_B_ receptor blocker, *n* = 4) groups. (c) TLR4 in the control, DNP, DNP + baclofen (GABA_B_ receptor agonist), and DNP + saclofen (GABA_B_ receptor blocker) groups. ^∗^*p* < 0.05; ^∗∗^*p* < 0.01.

Western blotting revealed that expression of Myd88 (*p* < 0.01, Figure 4(a), *n* = 6) and NF-*κ*Bp65 (*p* < 0.05, Figure 4(b), *n* = 6) was significantly increased in the spinal cord of the DNP group (*n* = 4) compared with those in the control group (*n* = 4). Administration of LPS-RS ultrapure (TLR4 antagonist, i.t.) significantly decreased expression of Myd88 (*p* < 0.05) and NF-*κ*Bp65 (*p* < 0.01) in the DNP + LPS-RS group (*n* = 6) compared with that in the DNP group. Furthermore, i.t. injection of MIP (a Myd88 antagonist) significantly decreased the expression of NF-*κ*Bp65 (*p* < 0.01) in the DNP + MIP group (*n* = 6) compared with the DNP group.

### 3.5. Effects of GABA_B_ Receptor Activation or Blockade on TLR4, Myd88, and NF-*κ*B Expression

**Figure 5:**
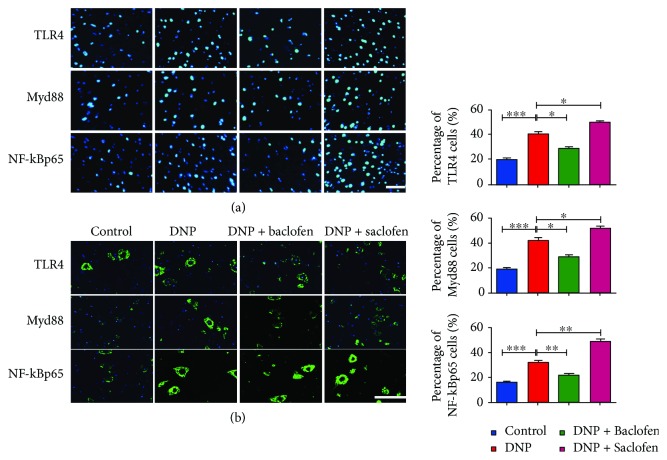
Single fluorescence labeling of TLR4, Myd88, and NF-*κ*Bp65 (nucleus stained with DAPI) in the spinal cord of the control, DNP, DNP + baclofen, and DNP + saclofen (*n* = 4) groups. Quantification of the percentage of TLR4, Myd88, and NF-*κ*Bp65-positive cells relative to all cells. Bar graphs represent mean ± SD. Scale bars are 100 *μ*m in (a) and 50 *μ*m in (b). ^∗^*p* < 0.05; ^∗∗^*p* < 0.01; ^∗∗∗^*p* < 0.001.

Western blotting and immunofluorescence revealed that TLR4 (*p* < 0.01, *p* < 0.001, Figure 4(c)), Myd88 (*p* < 0.05, *p* < 0.001), and NF-*κ*Bp65 (*p* < 0.01, *p* < 0.001) expression was significantly upregulated in the spinal cord of the DNP group compared with those in the control group. GABA_B_ receptor activation greatly downregulated expression of TLR4 (*p* < 0.01, *p* < 0.05), Myd88 (*p* < 0.05), and NF-*κ*Bp65 (*p* < 0.05, *p* < 0.01) in the DNP + baclofen group compared with those in the DNP group. GABA_B_ receptor blockade significantly upregulated expression of TLR4 (*p* < 0.05), Myd88 (*p* < 0.05), and NF-*κ*Bp65 (*p* < 0.05, *p* < 0.01) in the DNP + saclofen group compared with those in the DNP group (*n* = 4) (Figures 4 and 5).

### 3.6. Changes in IL-1 and TNF-*α* in the Spinal Cord Based on ELISA

**Figure 6:**
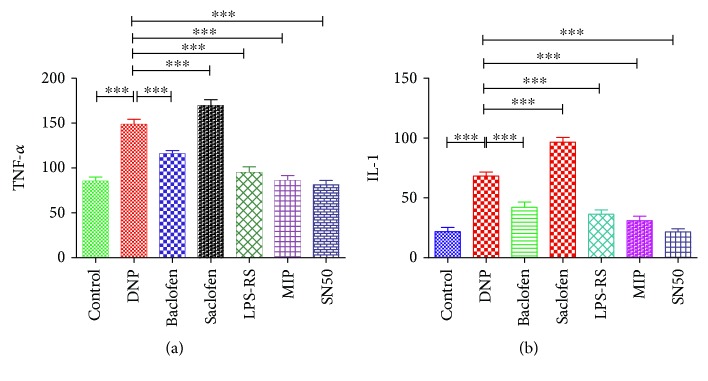
The expression of TNF-*α* and IL-1 in the spinal cord of control (*n* = 4), DNP (*n* = 4), DNP + baclofen (GABAB receptor agonist, *n* = 4), DNP + saclofen (GABAB receptor blocker, *n* = 4), DNP + LPS-RS (TLR4 antagonist, *n* = 6), DNP + MIP (Myd88 antagonist, *n* = 6), and DNP + SN50 (NF-*κ*B inhibitor, *n* = 12) groups by ELISA. (a) TNF-*α*. (b) IL-1. ^∗∗∗^*p* < 0.001.

ELISA showed significantly increased expression of IL-1 and TNF-*α* in the spinal cord of the DNP, DNP + baclofen, and DNP + saclofen groups (*n* = 4, *p* < 0.001) compared with those in the control group. GABA_B_ receptor activation or separate inhibition of TLR4/Myd88/NF-*κ*B signaling pathway both significantly decreased IL-1 and TNF-*α* expression in the spinal cord of the DNP + baclofen (*n* = 4), DNP + LPS-RS (*n* = 6), DNP + MIP (*n* = 6), and DNP + SN50 groups (*n* = 12) compared with those in the DNP group (*p* < 0.001). GABA_B_ receptor blockade significantly upregulated the expression of IL-1 and TNF-*α* in the spinal cord of the DNP + saclofen group (*n* = 4, *p* < 0.001) compared with those in the DNP group (Figure 6).

## 4. Discussion

In this study, we observed the upregulation of TLR4, Myd88, NF-*κ*Bp65, IL-1, and TNF-*α* expression in the spinal cord in DNP rats, accompanied by significantly decreased PWT and PWTL compared with normal rats. These findings are consistent with a previous report [14]. LPS-RS ultrapure, a TLR4-selective antagonist, downregulated downstream MyD88, NF-*κ*Bp65, IL-1, and TNF-*α* expression in the spinal cord of DNP rats and relieved pain. Both MIP (Myd88 antagonist) and SN50 (NF-*κ*B inhibitor) alleviated DNP and downregulated the expression of downstream signal factors. Overall, our findings suggest that activation of the TLR4/MyD88/NF-*κ*B signaling pathway, with activated microglia and astrocytes boosting the release of proinflammatory factors, was closely related to the development and maintenance of DNP. In addition, our results imply that inhibiting TLR4/Myd88/NF-*κ*B signaling pathway had analgesic effects in DNP rats.

DNP is a type of neuropathic pain. Neuropathic pain has common etiology, that is, peripheral nerve injury caused by different reasons including hyperglycemia, chemotherapy, mechanical pressure, or inflammatory activation [22]. Furthermore, peripheral nerve injury induces spinal microglia/astrocytic activation, which can release many substances including proinflammatory cytokines that intensify pain transmission by neurons [23, 24]). Proinflammatory factors, such as saturated fatty acids (SFAs), activate spinal microglia via the TLR4/NF-*κ*B signaling pathways [25, 26]. The Toll-like family, including TLR2 and TLR4, is correlated to innate immune responses and is also related to adaptive immunity [27, 28]. TLR2/4, especially TLR4, links microglial activation and nerve injury, which play an important role in the occurrence of neuropathic pain [12, 29].

TLRs are extensively distributed in the central and peripheral nervous systems, including in glial cells and neurons. As an important member of the TLR family, TLR4 is mainly located in primary sensory neurons, microglia, and astrocytes [29]. Previous studies have reported that TLR4 is upregulated in the spinal cord and dorsal root ganglion in paclitaxel-induced neuropathic pain, which is considered to have analogous mechanisms with DNP [30]. The TLR4/Myd88/NF-*κ*B signaling pathway and downstream inflammatory factors, including IL-1 and TNF-*α*, play a key role in the process of neuropathic pain [31–33]. Both TLR4 and CXCR4 are G protein-coupled receptors and can bind to the HMGB1 in cytoplasm, which expression level is elevated in diabetes [34, 35]. The binding of TLR4 and CXCR4 to HMGB1 is crucial to trigger chronic inflammation response in diabetes [36, 37]. With binding to HMGB1, TLR4 can promote the release of cytokines [38], while CXCR4 binding to HMGB1 increases the release of chemokines [39]. Both TLR4 and CXCR4 are abundant in glial cells in superficial dorsal horn in the spinal cord, which are important sensory regulation center that are closely related to the occurrence of neuropathic pain [40]. In the neuropathic pain model caused by partial sciatic nerve ligation, chronic postischemia pain, and spinal cord injury, TLR4/MyD88/NF-*κ*B and CXCL12/CXCR4 pathways are both activated and play important role in mediating pain transmission [41, 42]. Moreover, downregulation of CXCL12/CXCR4 inhibits glial TLR4 activation in the spinal cord and alleviates pain in the ischemia-reperfusion-induced inflammatory pain model [40]. Collectively, these findings suggest the interaction between TLR4/MyD88/NF-*κ*B signaling pathways and CXCR4 plays an important role in the diabetic neuropathy. Consistently, upregulation of TLR4 during the development of DNP in the spinal cord has been identified, and several drugs including coenzyme Q10 and duloxetine contribute to TLR4/Myd88/NF-*κ*B signaling pathway inhibition in the occurrence of neuropathic pain [14, 43, 44]. However, there is still a lack of evidence for the specific roles of the TLR4/Myd88/NF-*κ*B signaling pathway in DNP. The current study identified that the occurrence of DNP was closely related to the upregulation of the TLR4/Myd88/NF-*κ*B signaling pathway, in accordance with previous research.

In this study, we intrathecally administrated baclofen to examine the effects of GABA_B_ receptor activation on TLR4/MyD88/NF-*κ*B signaling pathways. In the DNP + baclofen group, mechanical allodynia and thermal allodynia were significantly attenuated and the expression of TLR4, MyD88, NF-*κ*Bp65, and downstream IL-1 and TNF-*α* in the spinal cord was downregulated, compared with those in the DNP control group. In the DNP + saclofen group, PWT and PWTL decreased and the expression of TLR4, MyD88, NF-*κ*Bp65, and downstream IL-1 and TNF-*α* in the spinal cord was upregulated compared with those in the control DNP group. Therefore, our study provides new functional evidence that spinal GABA_B_ receptors interact with TLR4/MyD88/NF-*κ*B signaling pathways in DNP rats, and the analgesic effect of GABA_B_ receptor agonist treatment is related to the inhibition of TLR4/MyD88/NF-*κ*B signaling pathways and downstream inflammatory factors in the spinal cord of DNP rats.

GABA_B_ receptors are G protein-coupled metabotropic receptors which are widely distributed in the central nervous system, especially in the spinal cord dorsal horn [45]. The primary role of GABA_B_ receptors in the central nervous system is to function as autoreceptors for feedback regulation of synaptic GABA release. Presynaptic and postsynaptic GABA_B_ receptors also inhibit synaptic transmission through regulation of synaptic glutamate release and promoting potassium channel opening [4, 6]. Furthermore, modulation of GABA_B_ receptors in the immune system suggested that GABA is a regulator of immune cell activity and inflammation [17, 18]. *In vivo* and *in vitro* research has demonstrated that activation of GABA_B_ receptors protected neurons from apoptosis and prevented transient focal cerebral ischemia [46, 47]. Activation of GABA_B_ receptors downregulated TLR4-induced NF-*κ*Bp65 nuclear expression and proinflammatory cytokine release in mixed glia in a multiple sclerosis (MS) model [18]. Baclofen and several other GABA_B_ receptor antagonists are allosteric modulators of CXCR4, a chemokine receptor that plays a key role in neuroimmune crosstalk [48]. Compelling evidence suggests that GABA_B_ receptors in the central nervous system regulate synaptic transmission through modulation of neurotransmitters release as well as neuroimmune regulation. In addition, our previous research demonstrated that expression of GABA_B_ receptors in the spinal cord was downregulated in DNP rats, and i.t. injection of the GABA_B_ receptor agonist baclofen in DNP rats produced antinociception [15]. Moreover, in paclitaxel-induced neuropathic pain, which has similar mechanisms with DNP, baclofen downregulated the expression of NF-*κ*B and alleviated DNP [17]. We confirmed the role of neuroinflammation in the occurrence of DNP, which was in accordance with the previous study. Furthermore, we demonstrated an interaction between GABA_B_ receptors and the TLR4/MyD88/NF-*κ*B signaling pathways in the spinal cord of DNP rats.

In summary, the present research suggested that TLR4/Myd88/NF-*κ*B signaling pathways play an important role in the occurrence of DNP. Our study reported for the first time that activation of GABA_B_ receptors in the spinal cord downregulated the expression of factors in the TLR4/Myd88/NF-*κ*B signaling pathway and alleviated neuronal inflammation, which is closely related to the analgesic effects of GABA_B_ receptor agonists.

### 4.1. Perspectives

In this study, we found the interaction between GABA_B_ receptors and TLR4/Myd88/NF-*κ*B pathwaysin the spinal cord in diabetes neuropathy. However, the signaling pathways that mediate GABA_B_ receptors and TLR4 remain unclear and warrant further investigation. On the other hand, if the activation of NF-*κ*B and the associated increase in inflammatory factors can influence the balance between apoptosis and autophagy in the central nervous system, the synaptic plasticity induced by NF-*κ*B and inflammatory factors may need to be elucidated in future studies.

## Figures and Tables

**Table 1:** Comparison of mechanical paw withdrawal threshold (in grams, *n* = 12, mean ± SE) in rats.

Group	Baseline	7 days	14 days	21 days
Control	14.17 ± 2.85	13.87 ± 2.58	14.86 ± 2.69	14.26 ± 2.63
DNP	14.86 ± 2.33	12.42 ± 1.92	4.78 ± 1.45^∗^^,^^∗∗^	4.28 ± 1.45^∗^^,^^∗∗^
LPS-RS + DNP	13.88 ± 3.03	11.89 ± 3.22	4.82 ± 1.32^∗^^,^^∗∗^	4.42 ± 1.38^∗^^,^^∗∗^
SN50 + DNP	14.98 ± 1.98	13.12 ± 2.76	4.67 ± 1.21^∗^^,^^∗∗^	4.51 ± 1.55^∗^^,^^∗∗^
MIP + DNP	15.07 ± 2.42	12.32 ± 2.97	4.77 ± 1.44^∗^^,^^∗∗^	4.62 ± 1.48^∗^^,^^∗∗^
Baclofen + DNP	14.55 ± 2.66	13.65 ± 3.48	5.14 ± 1.53^∗^^,^^∗∗^	4.84 ± 1.50^∗^^,^^∗∗^

^∗^Compared with baseline values, *p* < 0.05. ^∗∗^Compared with the control group, *p* < 0.05.

**Table 2:** Comparison of thermal paw withdrawal latency (in seconds, *n* = 12, mean ± SE) in rats.

Group	Baseline	7 days	14 days	21 days
Control	15.6 ± 2.8	115.8 ± 2.9	15.7 ± 2.7	15.9 ± 2.6
DNP	15.8 ± 3.5	111.8 ± 3.5	8.4 ± 2.5^∗^^,^^∗∗^	7.8 ± 1.7^∗^^,^^∗∗^
LPS-RS + DNP	16.9 ± 3.3	112.6 ± 3.9	7.9 ± 1.9^∗^^,^^∗∗^	7.2 ± 2.0^∗^^,^^∗∗^
SN50 + DNP	16.2 ± 2.9	113.5 ± 4.0	8.2 ± 2.1^∗^^,^^∗∗^	7.5 ± 2.1^∗^^,^^∗∗^
MIP + DNP	15.9 ± 2.6	112.9 ± 3.8	7.7 ± 1.6^∗^^,^^∗∗^	7.7 ± 2.3^∗^^,^^∗∗^
Baclofen + DNP	16.5 ± 3.8	113.3 ± 4.4	8.1 ± 2.2^∗^^,^^∗∗^	7.9 ± 1.6^∗^^,^^∗∗^
Saclofen + DNP	15.5 ± 2.7	112.7 ± 3.6	7.5 ± 1.8^∗^^,^^∗∗^	7.2 ± 2.2^∗^^,^^∗∗^

^∗^Compared with baseline values, *p* < 0.05. ^∗∗^Compared with the control group, *p* < 0.05.

## Data Availability

The data including behavioral testing data, biochemistry assay data, and immunohistochemistry data used to support the findings of this study are available from the corresponding author upon request.
